# Tailored cure profiles for simultaneous reduction of the cure time and shrinkage of an epoxy thermoset

**DOI:** 10.1016/j.heliyon.2024.e25450

**Published:** 2024-02-01

**Authors:** Jesper K. Jørgensen, Lars P. Mikkelsen

**Affiliations:** Department of Wind and Energy Systems, Technical University of Denmark, Roskilde, DK-4000, Denmark

**Keywords:** Cure kinetic, Thermosets, Finite element, Digimat, Abaqus

## Abstract

Defining the specific cure profile of thermosetting polymers is an important aspect in many applications where the mechanical performance and appearance of components can be affected. Cure-induced strains or stresses from the shrinkage of thermosets lead to reduced performance due to accelerated damage or discarded products due to distortions. This research focuses on validating a proposed modelling framework, simulating the load-transferring part of the curing process affecting the mechanical performance. The model's accuracy is evaluated against experimental results, and the model prediction is found to be within an accuracy of 2-8% of the experimental results. A 16-hour and 31-hour two-stage cure profile was compared and validated experimentally. The short profile results in a higher cure-induced of -0.56% with the longer profile yielding -0.46% cure-induced strain. Based on the model, a new three-stage cure profile has been proposed. Using this, it is possible to achieve a low level of cure-induced strain of -0.45% at a shorter cure time on 18 h.

## Nomenclature

α: Thermal expansion
Isothermal - Pre-cure
Heat-up before post-cure
Isothermal - Post-cure
Cool-down after post-cure
Heat-up before Pre-cure
Isothermal - Initiation-cure
Δ: Increment
$\varepsilon^{tot},\varepsilon^{th},\varepsilon^{ch}$: Strain (total, thermal and chemical)
$A_{1},A_{2}$: Pre-exponential parameter
$e_{a1},e_{a2}$: Activation energy
$K_{1},K_{2}$: Arrhenius function
m,n: Power law parameter
R: Universal gas constant
T: Temperature
t: Time
Vshend: Total load transferring volumetric shrinkage for $X\geq X_{\sigma }$
Vsh: load transferring specific volumetric shrinkage
X: Degree of cure
$X_{\sigma }$: Degree of cure - At load transfer initiation
Xend: Degree of cure - End condition considered as fully cured

## 1 Introduction

Thermosetting polymers are used in a wide range of industries across multiple sectors with different applications in mind. Either applied directly as, e.g. moulding compounds, coatings, adhesives or in the manufacturing of fibre composites. Epoxy, polyesters and vinyl esters are examples of thermosets employed within these manufacturing applications. Regardless of the area of application, it is of interest to understand the curing of these thermosets as well as predict their behaviour, by applying different temperature profiles. It is known that the curing process can lead to severe distortions and residual stresses. These residual stresses can lead to a shorter lifetime or even shed of components [1], [2], [3], [4]. In recent years, studies [3], [4], [5] have shown that the fatigue life of fibre composite materials is affected by the cure profile. The studies showed that using cure profiles and thereby introducing high residual stresses, significantly reduces the fatigue lifetime of the test specimens.

However, it is not only fibre composites where using a proper cure profile is relevant. Another large industry sector depending on thermosets is the electronic business where thermosets are used due to their physical properties and their resistance to heat. Hence, using thermosets to shield off printed circuit boards (PCB) with e.g. epoxy moulding compound (EMC) which is a mixture of epoxy and minerals. In the work by Hu et al. [2], the modelling of EMC for mounting PCB was studied. The paper dealt with predicting distortions due to the curing of the EMC. Distortions in this application are unwanted due to difficulty with mounting the PCB inside a manufactured shielding for the PCB.

There is a need to evaluate and propose techniques to reduce and limit defects from curing in thermosets. A way of gaining an understanding of the material behaviour is by in-situ monitoring during curing experiments. Here, different cure profiles at various temperatures can be tailored and compared to the amount of shrinkage, and hence strain is achieved in the material over time. Several methods for measuring the cure-induced strains have previously been proposed. Methods involving digital image correlation (DIC) for monitoring curing in-situ have been used by Kravchenko et al. [6] on composites. Another method has been the application of Fibre-Bragg-Gratings (FBG) for monitoring the strains from curing which has been applied in both composites and neat thermosets [7], [8], [9]. A method for in-situ measurement of curing in an epoxy system was proposed and tested with multiple different cure profiles to characterise and limit the strains induced. These methods of experimental testing are useful to get the knowledge of thermoset behaviour but can be time and labour-intensive.

To reduce and limit these testing methods, modelling could be a preferred option as properly validated models can be used to predict the curing process. Models used to predict the curing of thermosets have been proposed with the purpose of predicting cure-related physical distortions in EMC [2]. In another instance, where predicting the thermomechanical properties was the scope, a model was built and formulated by Rabearison et al. [10]. Both the mentioned papers discussed the importance of including the chemical nature and the thermal expansion of the polymer in the modelling phase while both papers stated that the viscoelastic behaviour during curing was not important for this purpose. The thermal expansion in both papers [2], [10] is assumed to depend on the glass transition temperature and includes that the thermal expansion above the glass transition temperature can be higher.

In order to capture the chemical-related changes in thermosets, several approaches have been applied over the years. The volumetric shrinkage was initially proposed in a model context by a relation from Bogetti and Gillespie [11]. The authors assumed a linear relationship between volumetric shrinkage and degree of cure. Furthermore, the authors proposed a model relating the incremental volumetric shrinkage to the incremental change of chemical strain. Other work was performed experimentally by Li et al. [12], where the resin was cured and the volumetric shrinkage monitored and reported as a function of the degree of cure. The non-linear dependency of the degree of cure was proposed by the authors to be interpreted as a bi-linear relationship where the inflexion point was the point of gelation. In this paper, the point of gelation was interpreted as the point where the chemical shrinkage is inhibited by the formation of molecular networks, and hence the shrinkage rate slows down. The authors reported a good prediction of the otherwise non-linear shrinkage before and after the gel point. This method was further used in a modelling context by Rabearison et al. [10]. Here, the part of volumetric shrinkage which occurs from the gel point until the material is fully cured was considered as a load-transferring shrinkage.

Another approach was the proposed shrinkage model by Johnston [13]. Here a second-order function for predicting volumetric shrinkage in an epoxy material was proposed. The relation was compared to experimental measurements and was reported to have a good correlation. The model proposed in [13] was an extension of a previously proposed linear shrinkage model [11].

The load-transferring part of the shrinkage in the polymer was investigated by a mechanical approach in [9]. The authors measured the in-situ shrinkage of the polymer through an optical fibre. Based on those measurements, the time and hence degree of cure at which the resin system was able to transfer deformation to the fiber was determined. This point was considered the load transfer point as it was a point from where the polymer was able to transfer deformation to the embedded optical FBG fibre.

In order to relate the volumetric shrinkage to the curing of the thermoset, a cure kinetic model is necessary. Many models have been proposed over the years, each with different aspects included to attempt improved cure prediction [14], [15], [16], [17]. One of the earliest models was proposed by Kamal [14], a model which since has been widely applied, due to its simplicity [10], [11], [18].

Johnston and Hubert [17] proposed a modified model based on the work origin by Cole et al. [16]. The model takes into account that for high degrees of cure, the reaction rate slows down due to vitrification of the polymer which occurs as the polymer enters a glassy state. The model proposed in these studies [16], [17] takes into account this change from kinetic-controlled chemical reactions to a diffusion-controlled reaction rate through the glass transition temperature.

The present study focuses on utilising an already existing framework for modelling the curing process. The focus will be on modelling a material point using a commercial finite element framework with the capability of later scaling into geometrically more complex models. The main scope of the work is to accurately predict the levels of cure-induced strain in a specific Epikote® RIMR 035c epoxy resin system with Epicure® RIMH 037 hardener. This will be achieved by taking into account the strain occurring both from the thermal expansion and the chemical volumetric shrinkage. The model will be validated against experimental results [9]. Furthermore, the model will be used for tailoring the cure profiles to reduce and improve the curing process of this specific thermoset system. The novelty is the development of a material point model capable of evaluating the behaviour of a cure profile before applying it in a manufacturing process.

## 2 Numerical modelling

### 2.1 Material constituents

The model aims to predict the curing process, taking the following points into account.

• The development of the degree of cure is based on an autocatalytic cure kinetic model.
• The chemical strain develops due to the changes in volume as a result of the polymerisation of the polymer in the load-transferring part of curing.
• A thermal strain develops due to the change of polymer volume due to the thermal expansion. The thermal expansion coefficient is assumed constant independent of the degree of cure.

The Constituents in (1), will lead to the following total strain

(1) $$\Delta \varepsilon^{tot}=\Delta \varepsilon^{ch}+\Delta \varepsilon^{th}$$

#### 2.1.1 Thermal strain

The thermal expansion of the material follows (2) which is

(2) $$\Delta \varepsilon^{th}=\alpha \Delta T$$

#### 2.1.2 Chemical strain

The load-transferring chemical strain in the model is defined as the incremental isotropic change in specific volumetric shrinkage (3)[11].

(3) $$\Delta \varepsilon^{ch}=\sqrt[{3}]{1+\Delta V_{sh}}-1$$

Where the incremental volumetric shrinkage, $\Delta V_{sh}$, is defined by the volume change of a cubic element with respect to its original volume and is thus, unitless. In this study, only the volumetric shrinkages that occur after the load transfer point are considered.

#### 2.1.3 Cure kinetic law

To predict the cure of the specific epoxy system, a well-established cure kinetic model (4), proposed by Kamal [14] will be used.

(4) $$\frac{d\;X}{dt}=(K_{1}(T)+K_{2}(T)X^{m}){\left(1-X\right)}^{n},K_{1}(T)=A_{1}\exp\left(-\frac{e_{1}}{RT}\right),\;K_{2}(T)=A_{2}\exp\left(-\frac{e_{2}}{RT}\right)$$

Here, *X* is the degree of cure and the derivative of this with respect to time, is the rate of cure. The temperature is denoted *T*, and the power law components are denoted *n* and *m*. The Arrhenius functions $K_{i}(T)$ depend on the pre-exponential factor $A_{i}$, the activation energy $e_{i}$ and the universal gas constant .

The parameters are found based on DSC measurements through a least-squares fit of (4) and tabulated in Table 1.

**Table 1:** Fitting parameter for the Kamal-Sourour model based on DSC from the Epikote® RIMR 035c epoxy resin system with Epicure® RIMH 037 hardener system from [9].

*A*_1_ [1/s]	*A*_2_ [1/s]	*e*_*a*1_ [J/mol]	*e*_*a*2_ [J/mol]	*n* [-]	*m* [-]
4.73 ⋅ 10^4^	3.15 ⋅ 10^5^	5.99 ⋅ 10^4^	5.71 ⋅ 10^4^	1.69	0.39

During the simulations, the degree of cure is integrated numerically (5) as

(5) $$X=\int_{0}^{t}\frac{\text{d}\;X}{\text{d}t}\text{d}t$$

The integrated *X* thus depend on temperature and time, relating the cure kinetic model to the specific curing profile applied to the polymer. The degree of cure *X* is directly related to the development of volumetric shrinkage required to express the chemical strain (3).

#### 2.1.4 Cure dependent load-transferring volumetric shrinkage

The cure-related shrinkage of the epoxy in the liquid phase will be ignored in the model. Liquid shrinkage cannot contribute to the development of residual stresses. As it is the residual stresses that impact the mechanical performance [1], [3], [4]. Therefore, the cure shrinkage in the liquid phase is ignored and the model will, therefore, only consider the load-transferring volumetric shrinkage. The load-transferring volumetric shrinkage as a function of *X* is a second-order model, Johnston [13].

(6) $$V_{sh}=\left\{ \begin{array}{cc} 0, & X<X_{\sigma }\\ V_{sh}^{end}{\left(\frac{X-X_{\sigma }}{X_{end}-X_{\sigma }}\right)}^{2}, & X_{\sigma }\leq X<X_{end}\\ V_{sh}^{end}, & X\geq X_{end} \end{array}\right.$$

**Figure 1:**
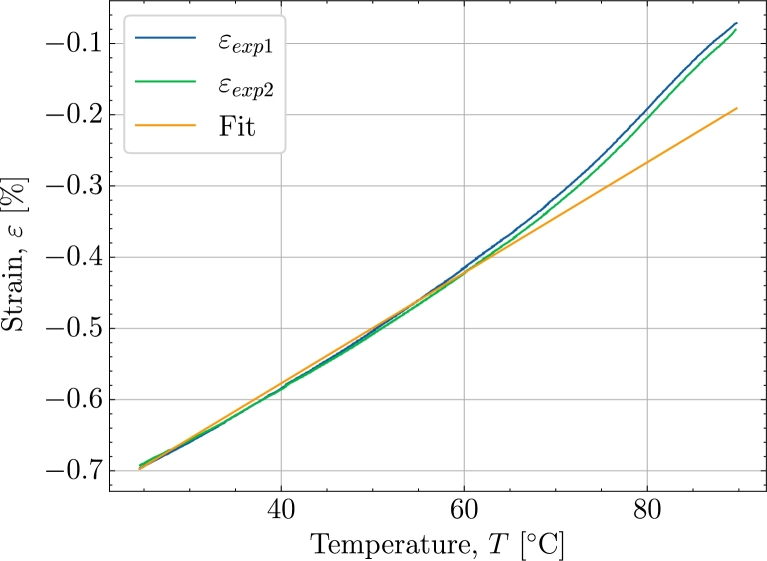
Reheating of fully cured panels in a range from 24 °C to 90 °C and the fitting of a thermal expansion coefficient in the range from 24 °C to 50 °C.

The load-transferring volumetric shrinkage $V_{sh}$ will develop when the cross-linking during curing has formed a network strong enough to transfer load. The load transfer initiation can be related to the degree of cure as $X_{\sigma }$ where subscript *σ* denotes the initiation of the load transfer point. The degree of cure, at load transfer initiation, $X_{\sigma }$ is found experimentally based on a strain tolerance using an optical FBG [9]. The shrinkage model (6) follows that the load-carrying part of the shrinkage will happen from the point of gelation described as $X_{gel}$. Thereby, these simulations assume that the shrinkage from the load transfer point $X_{\sigma }$ found by [9] till the end of the cure will result in the final cure-induced strain. The load-transferring shrinkage will continue until the material is fully cured. However, for numerical reasons, an end condition defining the fully cured state is used in the model, denoted $X_{end}=0.99$ as X=1 is a theoretical limit that will never be reached in practice. The parameters, necessary to characterise the shrinkage related to chemical curing and the thermal expansion are tabulated in Table 2. The point of load transfer for this material was found to be $X_{\sigma }=0.75$ [9]. To determine the thermal expansion and the strain contributions coming from this, a fitted value in the temperature range from 24 °C of the thermal expansion has been determined based on measurements of a fully cured panel from earlier studies [19] reheated from 22 °C to 90 °C with an embedded strain sensor. Fig. 1 shows the reheating and the fitted thermal expansion coefficient shown in Table 2. Based on $\varepsilon^{ch}=-0.39\text{\%}$ found in [9], the total volumetric shrinkage in the load transferring part of the cure $V_{sh}^{end}$ is estimated from (3).

**Table 2:** Shrinkage parameters for the Johnston shrinkage model and thermal expansion applied in the modelling [13].

*α* []	*X*_*σ*_ [-]	*X*_*end*_ [-]	$V_{sh}^{end}$ [-]
7.76 × 10^−5^	0.75	0.99	0.01175

The development of the cure-dependent load transferring shrinkage over time can be related to the incremental chemical strain in (3) by deriving (6) to incremental form by differentiation with respect to *X* and *t* as in (7). This is to have the volumetric shrinkage in the incremental form for the modelling perspective.

(7) $$\Delta V_{sh}=\frac{\text{d}\;V_{sh}}{\text{d}X}\Delta X\;;\;\;\Delta X=\frac{\text{d}\;X}{\text{d}t}\Delta t$$

### 2.2 Simulation methodology

The simulation methodology is based on some decisions related to the prediction of the cure strains. These decisions are compiled below.

– The model is defined as a material point where the cure behaviour is observed in a single node in the finite element model.
– A known temperature profile is used as input. This can be either generic or imported from experiments.

**Figure 2:**
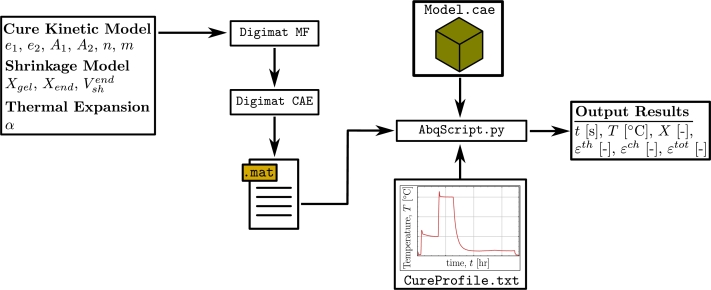
Flowchart of the coupled simulation framework between Digimat®and Abaqus®.

The modelling workflow is implemented with the means of handling input and output through Python scripting. A visual representation of the workflow with the simulation is given in Fig. 2. The material parameters from subsection 2.1 will be used as input and processed by the material software Digimat to build a UMAT file handled by Abaqus®. The Digimat part of the workflow is divided into two modules.

*Digimat® - MF*  A module able to perform Mean-Field homogenisation, thus abbreviated MF. In this simulation, the MF module is used to create an input file storing the material parameters used in the simulation [18].

*Digimat® - CAE*  The Computer-Aided-Engineering (CAE) module will receive the material parameters from the file created in MF. The output from Digimat CAE is a data file that is called by the Abaqus® simulation. Abaqus® invokes a UMAT (user-defined subroutine) that is a built-in part of the Digimat® software. The setup of the material definition used by the Digimat UMAT is only necessary to do once for a given material system. As the simulation is, in essence, performed only in a material point, the Abaqus finite element model is very simple. The model contains one 3D 8-nodal brick element (Model.cae) and is run by a Python script (AbqScript.py). In the script, the material setup follows an ordinary procedure for UMAT modelling as the remaining data comes from Digimat. The temperature profile (CureProfile.txt) is used as input and defines the simulation temperature and the simulation period. Post-processing is performed by the script writing the results to a file as shown in Fig. 2. The outputs are; time *t*, temperature *T*, degree of cure *X*, thermal strain $\varepsilon^{th}$, chemical strain $\varepsilon^{ch}$ and total strain $\varepsilon^{tot}$ for all increments. The script is available as an open-access Python script [20].

## 3 Results from validation

In order to conclusively use the model for predicting the load-transferring strains developing during the curing of the investigated Epikote® RIMR 035c epoxy system, the model will have to be validated in the following.

The validation process is based on a comparison with neat resin experiments [9]. The polymer in the considered validation experiment is free to deform in all directions, and thus no stresses will be present. In Table 3, five experimental cure profiles from [9] are tabulated. The profiles are based on temperatures measured inside a neat resin sample over a period of time. The two first cure profiles, *V1* and *V2*, are selected for validation as they are both at the same pre-cure and post-cure temperatures. The difference between the two profiles is the time duration of the pre-cure and post-cure. These profiles are used to investigate the model performance on a long cure time of 23 hours compared to one that is relatively short of 10 hours. The total cure time is defined as the sum of the isothermal time periods plus the heating ramps. The cool-down time after the post-cure is excluded from the reported cure time as the curing of the material can be considered ended before that part. The degree of cure at the end of the cure profiles is reported in the table as $X_{end}$.

**Table 3:** Cure profiles applied for the validation of the model.

Cure ID	Heat rate	Pre-cure	Heat rate	Post-cure	Total cure time	*X*_*end*_
	[K/min]	[h @ °C]	[K/min]	[h @ °C]	[h]	[-]
*V1*	1	12 @ 40	1	10 @ 80	23	0.995
*V2*	1	4 @ 40	1	5 @ 80	10	0.985
*V3*	1	20 @ 40	1	10 @ 80	31	0.995
*V4*	1	5 @ 50	1	10 @ 80	16	0.994
*V5*	1	2.5 @ 60	1	5 @ 80	8.5	0.989

In order to keep clarity in the visualisation, all plots will refer to specific regions on the cure profile. The following annotations are applied to the plots;

Isothermal - Pre-cure.
Heat-up before post-cure.
Isothermal - Post-cure.
Cool-down after post-cure.

### 3.1 Validation of *V1*

**Figure 3:**
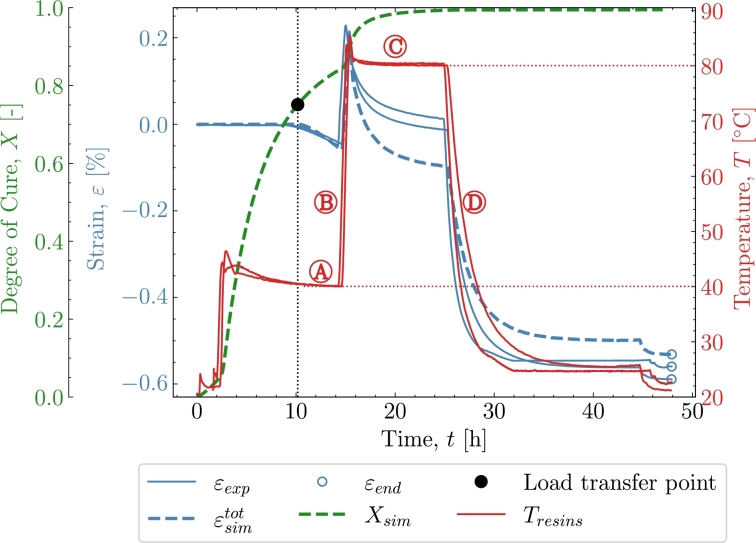
*V1* - Curing as a function of time for both experiment and simulation. **Note: that two similar experiments have been included.**

**Figure 4:**
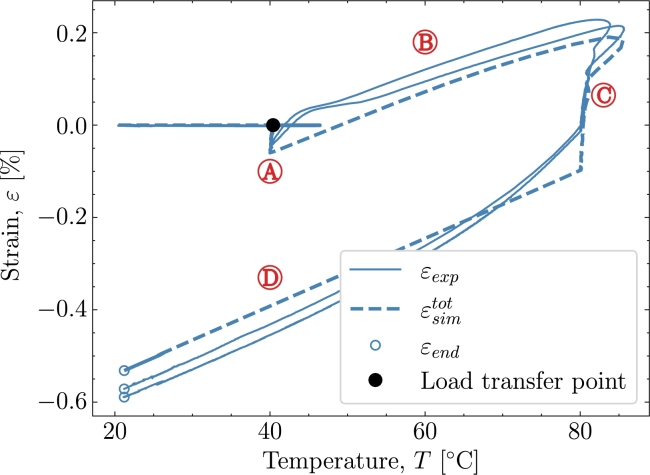
*V1* - Cure-dependent strain development as function of temperature.

Fig. 3 shows the results from the validation case of *V1*. The cure profile includes the exothermal behaviour combined with some temperature overshoot by the oven [9]. The combined exothermal and oven overshoot is observed by the temperature at the beginning of both isothermals  and . Two experimental datasets were available for this case; hence, both will be included in *V1* to show the variation of the experiments in context to the model. Fig. 3 shows the cure profile where strain development of experiment $\varepsilon_{exp}$ and simulation $\epsilon_{sim}^{tot}$ is plotted as a fucntion of time. The degree of cure $X_{sim}$, is simulated based on the parameters from subsection 2.1 where (4) has been numerically integrated. The black dot marks the load transfer point, which, in this case occurs after approximately 10 hours of curing associated with the defined $X_{\sigma }=0.75$ from Table 2. The load-transferring cure-induced strains are seen to develop after $X_{\sigma }$. The strain development begins during the isothermal pre-cure part . The strain will in that part develop as a chemical shrinkage strain. The thermal expansion kicks in and the total load-transferring strains increase as seen in  during the subsequent polymer heat-up. When the polymer enters the post-cure isothermal part , the chemical strains continue to shrink but are now starting from a positive total strain value. A deviation of the prediction is found where a higher contraction is predicted than measured. Entering the cool-down  regime, *X* is no longer developing significantly. The strains, on the other hand, continue to drop significantly due to the thermal contraction. The value of the strain from both the experiment and simulation is taken at the end of the cool-down regime. Those values can be found later in Table 4. Fig. 4 shows the strain development and its dependency to the temperature *T*. The heat-up  and cool-down  are easily distinguished from the rest of the response as the expansion dominates these two phases. The isothermal part at pre-cure  and at post-cure  shows a response at a constant temperature, i.e., strains that appear due to factors not directly related to the heating and cooling of the polymer. The strains are evaluated from $X_{\sigma }$ until the end of the cure profile. The regions of the cure profile , ,  and  are once again marked to distinguish the areas in which they occur. Furthermore, the simulation's thermal and chemical strains are plotted here to further clarify their relation to the cure profile.

**Table 4:** End value of *ε* after the cure profiles at *T*_*room*_ = 21 °C .

ID	*V1*	*V2*	*V3*	*V4*	*V5*
*ε*_*exp*_ [%]	-0.582	-0.943	-0.513	-0.634	-0.674
$\varepsilon_{sim}^{tot}$ [%]	-0.535	-0.892	-0.532	-0.613	-0.675


Deviation [%]	8	6	4	3	1

**Figure 5:**
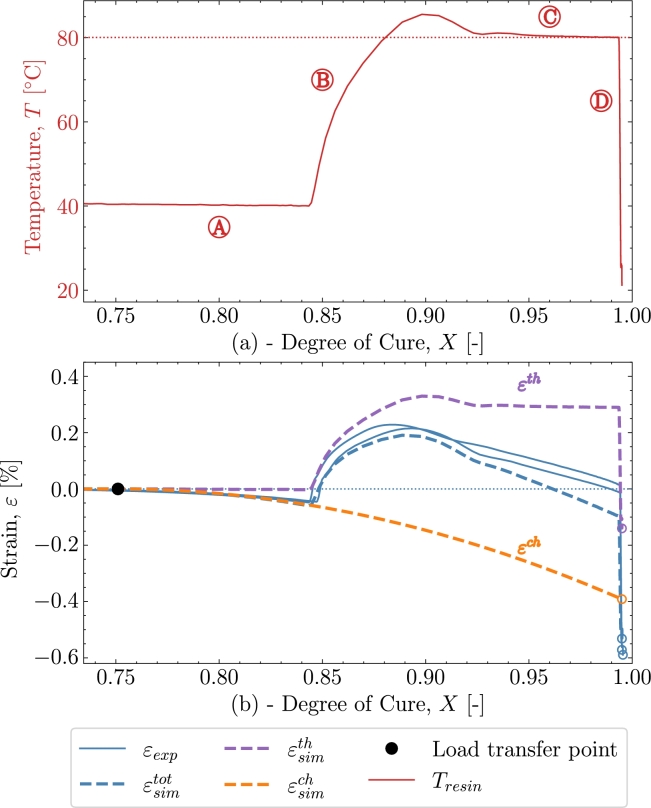
*V1* - (a) Temperature as function of cure *X* from point of load transfer. (b) Cure dependent strain development as function of cure *X*.

In the following, the simulation's behaviour with respect to the experiments will be discussed. Additionally, the time, temperature and chemical mechanisms present in the simulation and their ability to adapt the response in the experiment will be investigated. Fig. 4 is used to further support the cure behaviour illustrated in Fig. 3. The simulation is seen to underpredict the measurements in the early stages of . However, the overall response in this region is reasonable as the simulation and experiment's starting point and end point closely correlate during the heating in . Thus, the simulated thermal expansion is judged to be reasonable for the heat-up . Turning towards the cool-down , Fig. 4 shows that the strains from the experiment are dropping at a faster rate from 80 °C to 60 °C than from 60 °C to 40 °C indicating a non-constant thermal expansion coefficient as also observed in Fig. 1. In the simulation, however, this non-linear relationship is not taken into account as the thermal expansion is based on the measured expansion and a least-squares fit in the range from 24 °C to 50 °C and this leads to the apparent difference between the experiment and simulation. If one were to use the full non-linear curve in Fig. 1 the additional thermal contraction would correspond to 0.11%. Fig. 5 plot the strain now shown as a function of the degree of cure. The purpose of Fig. 5 is to show the strain developing as a function of the degree of cure and, thereby, that the volumetric shrinkage is uniquely defined by the degree of cure through (3) and (6) shown by the orange curve for $\varepsilon_{sim}^{ch}$. As $X_{\sigma }$ marks the initiation of the load transferring part of the chemical strain and because the initiation starts at the pre-cure isothermal , using Fig. 5 is ideal for separating the load-transferring cure-induced strain, dependent solely on *X*. As load transfer starts at a relatively constant temperature, there is a negligible contribution from the thermal expansion at this point in the curing and until the heat-ramp . This is reflected in a zero thermal strain $\varepsilon^{th}$ from $X_{\sigma }$ at the isothermal  until  begins. On the other hand, the chemical strain is developing during the isothermal part. This predicted chemical strain adapts the experiment rather well during the pre-cure . For the later post-cure isothermal , the deviation between the simulated total strain $\varepsilon^{tot}$, and the experimental strain indicates that for high values of *X*, the chemical strain is over-predicted. A possible explanation is that the cure model applied, is a pure autocatalytic model and this model does not take into account that the reaction slows down due to vitrification and hence that diffusion-controlled reaction rate is dominating when $T<T_{g}$ [17].

### 3.2 Validation of *V2*

**Figure 6:**
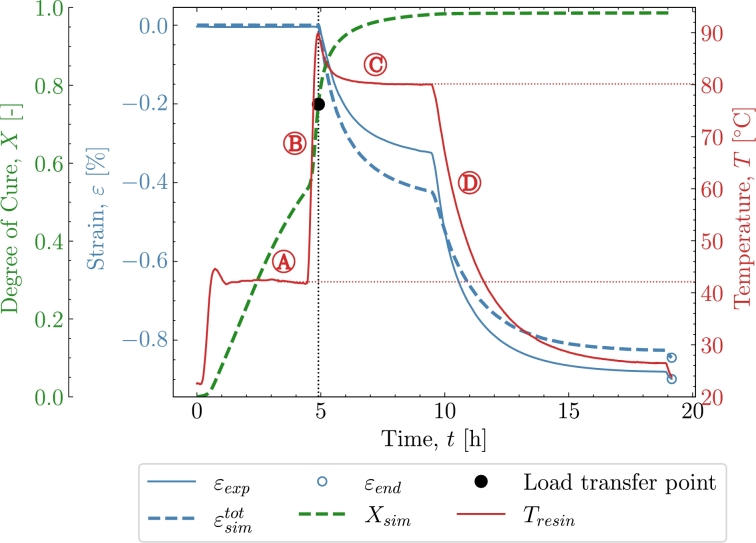
*V2* - Curing as a function of time for both experiment and simulation.

Fig. 6 shows an additional investigated case denoted *V2*. The cure profile is listed in Table 3 and is chosen as it is similar to *V1* apart from the significantly shorter cure time. Testing the model predictions when the load transfer initiation is at a different time and temperature than the first case. Fig. 6 also shows that the load transfer point is no longer achieved during the isothermal pre-cure temperature part, i.e. during part . Rather, this occurs during the ramp-up of the temperature , resulting in a load transfer point located at a fairly high temperature. Similar to the previous case the predicted strain also goes to a more negative level than the experimental measured value. Furthermore, the model deviates from the measurement during the temperature cool-down .

**Figure 7:**
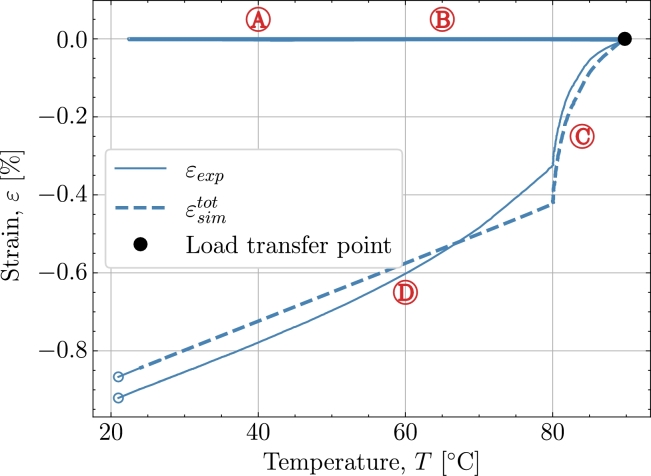
*V2* - Cure dependent strain development as function of temperature.

Fig. 7 shows the temperature dependency of the predicted strain in *V2*. Both during pre-cure isothermal  and heat up  no strains develop as the material is yet to reach load transfer. Close to the peak temperature, the load transfer point is reached. Despite the difference in strain development during the post-cure isothermal  the strain development during cool down  follows a similar tendency to *V1*. The model predicts a linear cool-down whereas the experiment is non-linear and thus deviates somewhat, especially at higher temperatures.

**Figure 8:**
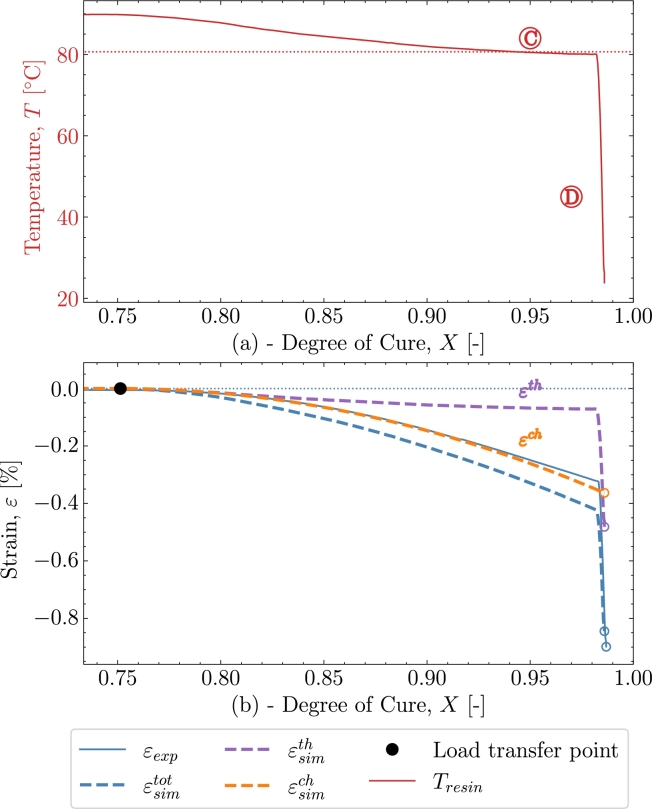
*V2* - (a) Temperature as function of cure *X* from point of load transfer. (b) Cure-dependent strain development as function of cure *X*.

Fig. 8 gives the strain development as a function of the degree of cure *X*. After the load transfer point the temperature settles at the prescribed 80 °C after an overshoot from around 90 °C . The load-transferring thermal strains developing in part  and  are small as seen in Fig. 8. Thus, the dominating strain developing is the chemical strain. Comparing the chemical strain to the experimental strain these follow each other during the isothermal . Hence, this indicates that the chemical strain is somewhat over-predicted in the model as the thermal strain is small and hence the dominating strain is the chemical strain. This is reflected in the total strain from the simulation $\varepsilon_{sim}^{tot}$ deviating from the experiment at .

Despite the differences between the experiment and the simulation the strain at the end of the curing profile is well predicted. The strains at the end of cure are compiled into Table 4 together with the results from 3 other validation cases V3, V4 and V5.

### 3.3 Discussion of validation

Evaluation of the simulation performed on the first validation case *V1* has shown that the simulation has drawbacks related to the behaviour of the thermal expansion where non-linear expansion present in the experiment is ignored in the model. Despite this, the thermal expansion is overall reasonable for the heat-up before post-cure. Although having a reasonable correlation at the early stages of curing, the chemical strains predicted for the model show that for high levels of *X*, the predicted strain deviates from the experiment. Taking these flaws into account the predicted strain at the end of cure was compiled in Table 4 where a number of additional cases were included. For *V1* the experiment shows $\varepsilon_{exp,end}=-0.582\text{\%}$. The simulated value of the total strain predicted at the end of the cure is $\varepsilon_{sim,end}^{tot}=-0.535\text{\%}$ and thus a lower cure-induced strain. In the end, the largest deviation observed between the experiment and simulation yields a difference of 8%. Taking into account the possible flaws described, the difference is relatively low.

For *V2* the experiment and model correlate well, similar to *V1*. Despite this, the non-linear expansion present in the experiment was not observed in *V2*. The model predicts a chemical strain that for high degrees of cure *X* is found to have some deviation. Although these deviations are observed, the end value of the strains found in Table 4 shows acceptable deviation for *V2* and hence the model predicts the final strain at the end of the cure time with reasonable accuracy. The two simulated experiments show that the cure-induced strains could be predicted within a reasonable accuracy. The directly observed deviations are due to the modelled behaviour, where the chemical strain is slightly overpredicted, and thermal contributions are slightly underestimated due to limitations of the linear thermal expansion model used.

Reasonable predictions have been found and hence the model can be considered as a valid tool for predicting the levels of load-transferring cure-induced strains developing for curing neat thermosets under different cure profile conditions. Applicable for planning cure profiles for limiting the cure time and/or cure-induced strains, which will be demonstrated in the following.

## 4 Results from tailored cure profiles

The previous validations of the model dealt with the comparison and simulation of two selected experiments. The model will in the following be used to predict and investigate the strains coming from the curing process and the influence of temperatures and time on the strain observed at the end of the cure profiles.

### 4.1 Tailoring of cure profiles

In order to predict the cure-induced strains and the final aim at minimising these, as well as the overall cure time, a selection of 3 cure profiles have been selected as tailored cases. These profiles are listed in Table 5. The profiles are based on the following scopes for evaluation.

*P1* – A profile with a high temperature at the pre-cure temperature to get a higher reaction rate and thereby a short cure time nevertheless, this results in high cure-induced strains at the end of the cure profile.
*P2* – A profile with a close to room temperature pre-cure to an expected lower cure-induced strain but now with an overall longer cure time.
*P3* – A profile, divided into 3 stages with an initial high temperature at cure to give a high reaction rate at the early stage. It is followed by a cool down to a low-temperature pre-cure. The material should reach its load-transferring state at a lower temperature to lower the contribution of thermal strains and thereby lower the cure-induced strains. Afterwards, a high post-cure temperature follows to cure the material fully. The expectation is to make a cure profile that will simultaneously give a shorter cure time with lower cure-induced strains.

The last cure profile listed is a three-stage cure, tailored to lower cure time and reduce strain obtained in the same process. The purpose of this is to lower the production time of components made from thermoset materials e.g. epoxy, and simultaneously limit the strains obtained from the load transfer point to the end of cure.

**Table 5:** Cure profiles applied for predicting cure-induced strains.

Cure ID	Heat rate	Pre-cure	Cooling rate	2. Pre-cure	Heat-rate	Post-cure	Total Cure time
	[K/min]	[h @ °C]	[K/min]	[h @ °C]	[K/min]	[h @ °C]	[h]
*P1*	1	6.5 @ 45	-	-	1	8 @ 80	15.5
*P2*	1	20 @ 30	-	-	1	10 @ 80	31
*P3*	1	3 @ 45	-0.1	3 @ 30	1	7 @ 80	17.5

The cure profiles adapted in these simulations are based on a user-defined input where the main parameters are listed in Table 5. These are based on the experimental observation and include the exothermal behaviour for the same resin system [9]. The implemented exothermal nature and oven overshoot in these simulations are mimicked based on the experimentally observed temperature profiles inside the resin system.

**Figure 9:**
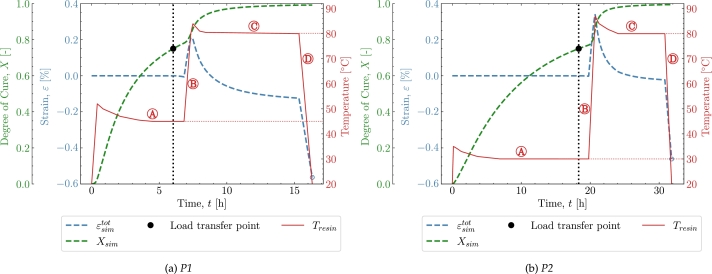
Cure profiles and simulations of *P1* and P2.

Fig. 9 shows the result of simulations based on *P1* and *P2* in Fig. 9a and Fig. 9b, respectively. For *P1*, the overall cure time should be relatively short but at the expense of a high cure-induced strain at the end of the cure. This is observed if one compares the final cure-induced strain with *P2* in Fig. 9b. The final cure-induced strains that arise in *P2* are observed to be significantly lower at the end of the cure profile. The cure profile *P2* will result in a lower strain by the end of the cure profile but with the cost of a significantly longer cure time. The reason for the quite different strains in those two cases is directly related to the temperature at which the load transfer initiates. The chemical nature of the thermoset is unavoidable. However, if the load transfer initiates at a low temperature, i.e. ideally as close to room temperature as possible, the thermal strains can be reduced. Thus, if one observes the load transfer point at the isothermal  in both *P1* and *P2*, the reason behind the difference in cure-induced strains is to be found there.

This illustrates the difficulties involved in selecting a 2-stage cure profile, as there are not as many degrees of freedom to manipulate the thermal contribution of the cure-induced strain. To overcome this, a smarter solution is to introduce a 3-stage cure profile. This allows for more freedom during the curing process. This can be achieved by first introducing an isothermal condition to accelerate the cure before the load transfer point. This will lead to the degree of cure evolving at a high rate in the early stages by in essence activating the exothermal behaviour. This state is referred to as the “Initial pre-cure”. Followed by a temperature drop to a second isothermal stage where one desires the load transfer point to be located. This state will be referred to as “Pre-cure”. This is followed by a heat up to a third isothermal stage where an ideally higher temperature will be held to cure the material fully - This state is referred to as the “Post-cure”. The cure profile *P3* in Table 5 is such a three-stage cure profile, tailored to achieve the behaviour explained above.

To assist the cure profile listed in Table 5 a letter annotation similar to the previously used system is adopted for explaining the cure profile.

Isothermal - Initial Pre-cure.
cool-down before pre-cure.
Isothermal - Pre-cure.
Heat-up before post-cure.
Isothermal - Post-cure.
Cool-down after post-cure.

**Figure 10:**
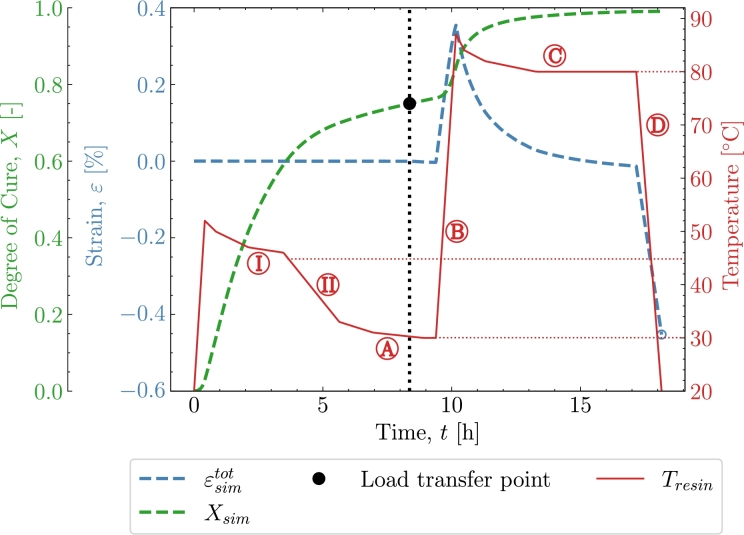
*P3* - Simulation of the tailored 3-stage cure profile in order to combine the advantage of short cure time and low cure-induced strains.

Fig. 10 shows a tailored 3-stage cure *P3*. The curing is initiated by a ramp-up at 1K/min to the first pre-cure isothermal of 45 °C denoted . An exothermal and oven overshoot is included based on experience from the oven and the resin system. The period of  is 3.5 h. The exothermal results in that the temperature does not reach 45 °C before the cool-down . The cool-down sequence is determined based on the possible cooling rate of an oven used for this purpose. The oven in question is only capable of cooling at 0.1 K/min. Hence a significantly long period of around 2 h is used before  is reached. It is assumed that as the material reaches the second pre-cure isothermal  the material will still behave slightly exothermal. Hence a small temperature overshoot is included, which decreases during this 3.5 h isothermal period at 30 °C. Load transfer is observed to initiate during this isothermal. After  follows the ramp-up  to the post-cure isothermal . A slightly smaller exothermal and oven overshoot is observed due to that most of the exothermal energy has been released during the initiation-cure . The post-cure is held for 6 h allowing the material to fully cure before the final cool-down .

**Figure 11:**
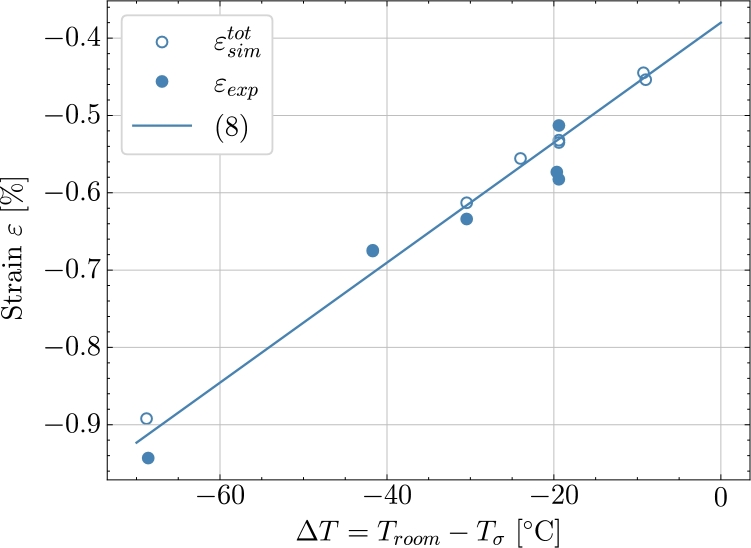
The curve based on (8) with  and *ε*^*ch*^ = 0.39% and the final cure-induced strain values from experiments *V1-V5* and simulations; *V1-V5, P1-P3*.

Fig. 10 illustrates that utilising a tailored 3-stages *P3*, instead of the previous 2-stage profiles *P1* and *P2*, makes a smart alternative between a long cure time with low cure-induced strain and short cure time with more cure-induced strain. The tailored profile applied in Fig. 10 achieves a smarter solution with a cure time similar to *P1* but with a similar magnitude of cure-induced strain as in *P2*. The cure-induced strains and cure time at the end of the cure profile for the three simulations *P1-P3* are found in Table 6. It is observed that *P2* and *P3* end with similar cure-induced strains but due to the long isothermal at 30 °C where the material cures very slowly the time for *P2* to be fully cured is almost twice of case *P3*. *P1* has a similar cure time but results in a larger cure-induced strain. As mentioned earlier, the behaviour of the material at 30 °C is possibly affected by the $T_{g}$ which for $T<T_{g}$ is known to slow down the cure rate significantly. The polymer enters a vitrified state that is diffusion-controlled and the chemical reactions slow down. For this epoxy, it can be found [9] that $T_{g}=22.95$ °C at X=0.75 and thus also at the load transfer point. This means that the influence on the predictions made with both *P2* and *P3* are not likely affected by the vitrification of the epoxy at the expected load-transfer point. For a condition of fully cured polymer, which is aimed to be $X_{end}=0.99$ in the model, the equation (8) can be used to define a relation of the total strain. The chemical strain for a fully cured material is $\varepsilon^{ch}=0.39\text{\%}$. The thermal expansion is known and inputted to the model, as listed in Table 2. Thereby, the equation can be used to check that the model gives the expected overall behaviour in terms of the level of thermal strain. Fig. 11 shows the experimental and simulated cure-induced strain values at the end of the cure. The included cases are the validation studies *V1-V5* as well as the predicted *P1-P3*. The plot shows that both the model and experiments follow the same trend as expressed by (8). Thus, confirming the observed increase in thermal strain due to the difference between room temperature and the temperature at the load transfer point.

(8) $$\varepsilon^{tot}=\alpha \left(T_{room}-T_{\sigma }\right)+\varepsilon^{ch}$$

**Table 6:** Cure Predictions - End value of strain after cure at *T*_*room*_ = 21 °C .

Cure ID	*P1*	*P2*	*P3*
*X*_*end*_	0.994	0.991	0.991
Predicted strain [%]	-0.563	-0.461	-0.453

## 5 Conclusion

This study aimed to predict the cure-induced strains in the curing process of a neat epoxy thermoset. A modelling approach was utilised and compared to an experimental setup. The modelling was approached through a finite element framework coupling two commercial software codes. The framework was reduced to observing the material behaviour at a material point level. The model was focused on capturing the strains developing during curing taking into account the chemical and thermal behaviour of the thermoset. The inputs for the chemical behaviour were based on a studied epoxy resin system and the thermal expansion was based on experiments.

In essence, the simulation methodology is neat and with few required inputs. The purpose of which, was to input the parameters of a given resin system and apply the model for prediction of the cure-induced strains. To ensure reasonable modelling accuracy, the model was validated against a range of experiments, with two experiments analysed in greater detail. In both of the cases studied, the overall observed predictions were reasonable. However, it was observed that there is room for improvement in capturing the thermal and chemical behaviour with this model hence narrowing the observed deviation window.

The model was tested on a prediction of two tailored cases with 2-stage cure profiles. The first case had a short cure time but with a resulting high cure-induced strain. The other had a longer cure time but a lower cure-induced strain. Not being able to achieve simultaneously, a short cure time and low cure-induced strain, were identified as the limitations in the 2-stage cure profiles as the pre-cure isothermal was where load transfer was initiated.

A third, and smarter 3-stage cure profile was tailored, to deliver a little thermal strain contribution induced in the end. The tailored profile delivered the best of the two other profiles i.e. a low amount of cure-induced strain in a short cure time period. This profile has the potential to positively affect production facilities where the cure time for thermosets often is a limiting factor. In addition, this can lead to components made from thermosets with reduced distortions and/or residual stresses.

## CRediT authorship contribution statement

**Jesper K. Jørgensen:** Writing – review & editing, Writing – original draft. **Lars P. Mikkelsen:** Writing – review & editing.

## Declaration of Competing Interest

The authors declare that they have no known competing financial interests or personal relationships that could have appeared to influence the work reported in this paper.

## Data Availability

The Python script used for modelling is available in [20]. Additional data can be made available on request.
